# High Mitochondrial Haplotype Diversity Found in Three Pre-Hispanic Groups from Colombia

**DOI:** 10.3390/genes14101853

**Published:** 2023-09-23

**Authors:** Daniel Uricoechea Patiño, Andrew Collins, Oscar Julián Romero García, Gustavo Santos Vecino, José Vicente Rodríguez Cuenca, Jaime E. Bernal, Escilda Benavides Benítez, Saray Vergara Muñoz, Ignacio Briceño Balcázar

**Affiliations:** 1 Doctoral Program in Biosciences, Human Genetics Group, Faculty of Medicine, University of La Sabana, Chía 250001, Colombia; daniel.uricoechea@unisabana.edu.co; 2 Human Genetics & Genomic Medicine, Faculty of Medicine, University of Southampton, Southampton SO16 6YD, UK; a.r.collins@soton.ac.uk; 3 Genetics Group, National Institute of Legal Medicine and Forensic Sciences, Bogotá 110311, Colombia; oscar.romero@medicinalegal.gov.co; 4 Department of Anthropology, Faculty of Social and Human Science, Universidad de Antioquia, Medellín 050010, Colombia; gsantosvecino@yahoo.es; 5 Research Group in Biological Anthropology, Universidad Nacional de Colombia, Bogotá 111321, Colombia; jvrodriguezc@unal.edu.co; 6 Faculty of Medicine, University of Sinú, Cartagena de Indias 130011, Colombia; jebernal@gmail.com (J.E.B.); escilda.benavidez@unisinu.edu.co (E.B.B.); sarayvergara@unisinu.edu.co (S.V.M.)

**Keywords:** ancient DNA, pre-Hispanic, mtDNA HVS-I, native American founding lineages, native American genetic history, native American ancestries, Colombia

## Abstract

The analysis of mitochondrial DNA (mtDNA) hypervariable region (HVR) sequence data from ancient human remains provides valuable insights into the genetic structure and population dynamics of ancient populations. mtDNA is particularly useful in studying ancient populations, because it is maternally inherited and has a higher mutation rate compared to nuclear DNA. To determine the genetic structure of three Colombian pre-Hispanic populations and compare them with current populations, we determined the haplotypes from human bone remains by sequencing several mitochondrial DNA segments. A wide variety of mitochondrial polymorphisms were obtained from 33 samples. Our results support a high population heterogeneity among pre-Hispanic populations in Colombia.

## 1. Introduction

The archaeological research conducted in Colombia has unveiled a history of habitation dating back to around 10,000 BP [1,2]. This timeframe has commonly been divided into three distinct periods: an initial phase characterized by hunter-gatherer societies, a subsequent era marked by pottery use, and a final epoch during which agricultural communities took root [3]. The latter phase corresponds to the indigenous peoples encountered by Spanish conquerors during the XVI century. These diverse groups, differentiated by their geographical locations, played a significant role in shaping the regional nomenclature of Colombia, contributing to designations such as “Bolo Temprano”, “Aburraes”, and “Laches”.

Recent archaeological discoveries propose three prehistoric periods in Colombia: the agro-potter period spanning from 500 to 900 BP (±100 years), the formative period from 1400 to 2800 BP (±200 years), and the Paleoamerican period extending from 5000 to 8000 BP (±2000 years) [4,5]. These models have undergone refinements and challenges through subsequent archaeological and genetic investigations [6,7,8,9,10].

Ancient DNA extraction and analysis from remains recovered at indigenous archaeological sites have emerged as vital tools for comprehending the origins of Colombia’s population. These endeavors strive to unravel the genetic makeup of pre-Columbian communities and, based on this genetic structure, elucidate genetic connections between these populations (Paleoindians) and the contemporary Colombian populace [11,12]. Additionally, the outcomes from these studies may offer insights into alternative genetic relationships, indicating scenarios of population replacements, migrations, and admixtures [2,11,13]. Consequently, the identification of crucial genetic insights holds the potential to provide essential guidance in constructing a coherent narrative about the settlement patterns of pre-Hispanic societies across the present-day territories of Colombia.

The unearthing of pre-Hispanic remnants within Colombia’s contemporary landscape unveils precious windows into the civilizations of antiquity that once thrived in the area. These archaeological revelations, encompassing not only human remains but also artifacts and architectural remnants, provide fascinating glimpses into the cultural traditions, societal structures, and pronounced genetic diversity of these ancient communities [13,14,15,16,17,18,19]. This holds immense significance, particularly when considering that these investigations delve into populations predating the era of Spanish conquest.

This study’s primary objective rested upon delineating the genetic makeup of three distinct pre-Hispanic population clusters in Colombia: the Bolo Temprano, Lache, and Aburrae groups. The overarching aim was to foster a deeper comprehension of the intricate phylogenetic interrelationships among these individuals. This exploration, in turn, offers a repository of molecular insights with the potential to illuminate the genetic essence of the pre-Columbian societies that once inhabited modern Colombian territories. Furthermore, it aims to illuminate the intricate genetic ties between these historic Native American population cohorts and the contemporary populace.

## 2. Materials and Methods

**Figure 1:**
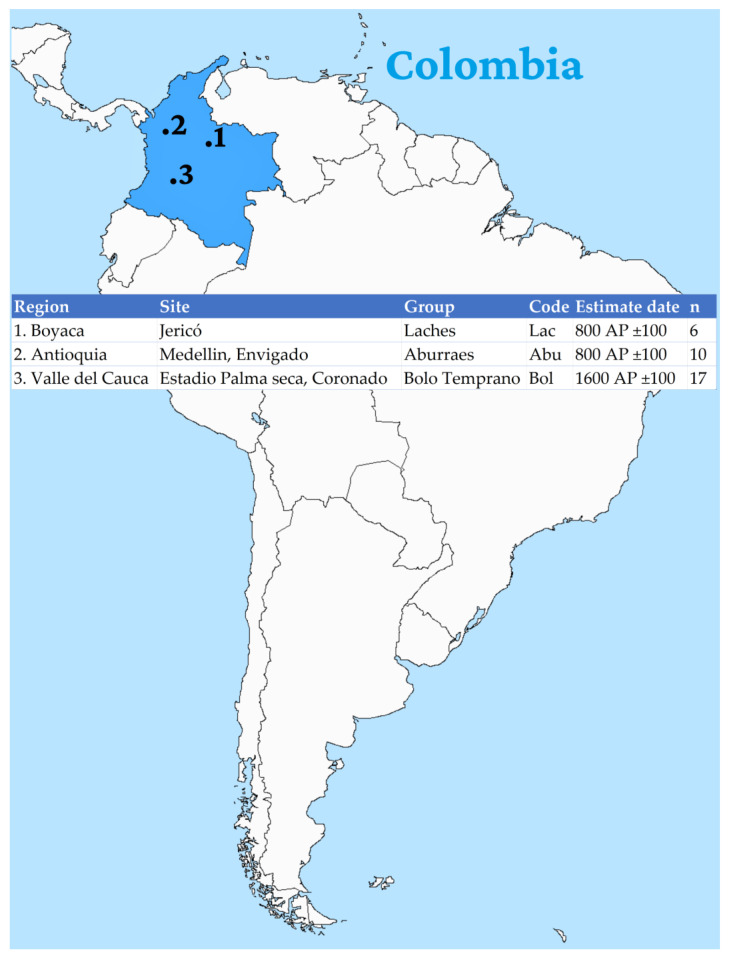
Displays the geographical distribution of ancient skeletal remains from a historical Colombian population. The figure provides information about the respective excavation sites, estimated dates, and the total number of samples that were comprehensively examined.

This study encompassed a total of 33 samples extracted from ancient remains associated with three distinct pre-Columbian indigenous groups [800–1600 AP]. Among these samples, six were sourced from the Lache community, recovered from sites within the Boyacá department’s Jericho region. Additionally, 10 samples originated from the Aburráe group, derived from individuals discovered in various locations within the Antioquia department, including Cerro del Volador (one sample), Cerro de la Colinita (one sample), Medellín, and the Alto de las Flores area (eight individuals). Lastly, 17 samples were linked to the Bolo Temprano population, originating from archaeological sites within the Valle del Cauca department (Figure 1).

To facilitate the analysis of these specimens, a human genetics laboratory was established at the Center for Biomedical Research, Universidad de la Sabana, Chía, Colombia, strictly adhering to the guidelines proposed by Hummel [20] to prevent sample contamination. The methodologies applied in this study were built upon previous research in ancient DNA analysis [21,22], tailored to the specific contexts.

The sample decontamination and treatment followed the procedures outlined by Casas-Vargas et al. [18], utilizing a tungsten carbide bur. For DNA extraction, the simple salting-out method was employed [15].

The determination of mitochondrial haplotypes centered on amplifying a 388 bp fragment from the hypervariable region I (positions 16,021 to 16,408) of human mtDNA, referencing the Cambridge Reference Sequence (rCRS) [23]. This amplification was carried out using four sets of PCR primers, designed to overlap within a 157–180 bp range, specifically tailored for pre-Columbian populations [24].

The PCR amplification process utilized 0.5 units of AmpliTaq Gold™ DNA Polymerase (hot start) from Applied Biosystems, 2 mM MgCl from Applied Biosystems, 1.25 mm dNTPs from Sigma, and 0.5 mM of each primer in a final volume of 1 μL, which was standardized to an extraction volume of 0.5 μL. PCR amplifications for each primer pair were performed using the Axygen^®^ MaxyGene™ THERM-1000 cycler, manufactured by Axygen (Union City, CA, USA), and housed at the Medical Research Science Center within the Faculty of Medicine at the University La Sabana in Chia, Colombia. The cycling conditions comprised an initial temperature of 95 °C for 8 min, followed by 40 cycles of 94 °C, 59.6 °C, and 72 °C for 1 min each. The amplified PCR products were validated through 2% agarose gel electrophoresis. Optimal bands were subjected to re-amplification in a final volume of 50 μL using the same conditions mentioned above. As part of the authenticity criteria, negative controls were included for DNA extraction and PCR [25].

For HVR I assembly and detection, CLC Genomics^®^ Workbench software, version 3.6.5, was employed. The program sequence (rCRS) was used as a template, determined after manual verification using Chromas software, version 2.5.0. To identify independent polymorphisms from extracts of the same sample, a consensus of at least two sequences was required. The determination of corresponding haplotypes involved manual cross-checks using EMPOP mtDNA database software, version v3/R11. The maximum likelihood method was applied for this estimation [26] and for individual haplotypes. The geographical locations of haplotypes were assigned, and a secondary confirmation was conducted utilizing HaploGrep^®^ 2.0 software [27,28,29]. To calculate genetic distances, Arlequin software, version 3.5.2.2, was employed. Additionally, a neutrality test and genetic variation indices were applied to individual sequences from the three populations. Likewise, comparisons were made between the haplotype frequencies in this study and those reported in previous investigations involving pre-Columbian population groups in Colombia [13,14,15,16,17,18,19]. UPGMA and neighbor-joining cluster analyses were conducted using the PHYLIP package, version 3.696. The resulting trees were generated using FigTree software, version 1.4. Furthermore, principal component analysis was performed using IBM SPSS Statistics software, version 23 [30,31].

Subsequent to a reevaluation of the mtDNA-based phylogenetic tree [32], several automated tools, such as Mitotool [28] and HaploGrep [29], were employed. This approach established a more dependable method for analyzing polymorphisms that required less data to accurately ascertain a haplogroup. The assignment of haplogroups entails aligning the mtDNA sequence with a reference sequence that encompasses a set of variants classifiable into corresponding global consensus-based haplogroups. This alignment is then cross-referenced with an open-access phylogenetic tree [32]. Similarly, a maximum likelihood estimation approach [33], using a typification database of 35,000 entries [27,32], facilitated the search for individuals worldwide who share similar mtDNA variations.

HaploGrep^®^ 2.0 software was employed to determine subhaplogroups and haplotypes, yielding values ranging between 0.55 and 0.77. Given the inherent nature of ancient DNA haplogroups and their correlation with the MITOMASTER tool in the MITOMAP database, which documents sequence variations in mtDNA, haplotypes within a similar range of values were utilized in this study.

## 3. Results

**Figure 2:**
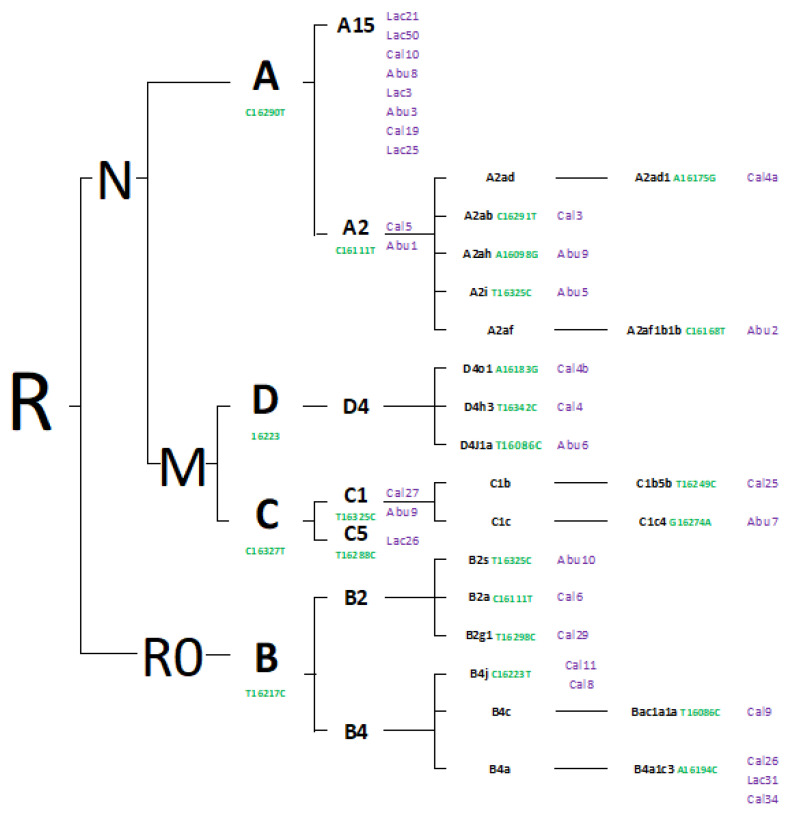
Depicts the phylogenetic tree with samples classified into haplogroups and haplotypes. All the sorted samples are amalgamated into a comprehensive tree encompassing all rCRS-related polymorphisms. Private polymorphisms known by Phylotree are indicated in green. The individuals are denoted in purple. Constructed through the neighbor joining algorithm based on Phylotree, the phylogenetic tree’s sample names have been abbreviated for clarity.

A total of 33 individuals underwent typing, leading to the identification of four founding haplogroups (A 45.455%, B 30.303%, C 15.152%, and D 9.09%) as well as 21 subhaplogroups. Furthermore, a phylogenetic tree was constructed to visually represent the evolutionary relationships that underscore a shared ancestral origin among the typified samples. Each individual was labeled according to the indigenous group they were affiliated with (Lac = Lache, Bol = Bolo Temprano, and Abu = Aburrae). The phylogenetic tree reveals a cohesive evolutionary lineage among these individuals, highlighting their common descent (shown in Figure 2).

The haplotypes of eight individuals assigned to haplogroup A had not been documented in the EMPOP, GenBank, and Mitomap databases prior to this study. Consequently, these specific haplotypes were excluded from the phylogenetic analysis [34].

Interestingly, while haplogroup A typically exhibits variations [G16319A] and [T16362C], these particular variants were not present in the eight individuals examined in the current study. Instead, all individuals in this study share variation [C16290T], which has been previously associated with haplogroup A. Notably, haplotype A2 demonstrates a widespread distribution across North America and is linked with a Native American haplogroup that is particularly prevalent in the northern latitudes of the continent [32]. This distribution encompasses Siberian tribes [35,36,37] who migrated from Asia to the Americas via the Bering Strait [38,39,40,41].

This affirmation finds validation in a mitogenomic analysis conducted by Achilli et al. [42]. Their study holds considerable significance in the realm of research involving ancient populations, particularly due to its relevance to the oldest samples dating back to 10,000 AP [43], which have been associated with haplogroup A2 [44]. Taking this evidence into consideration, it is plausible that haplogroup A2 may have made its way to the Americas during the initial migration waves. This notion is supported by its frequent presence in studies concerning pre-Hispanic populations [45], showcasing its extensive distribution across the continent [46].

Within the confines of haplogroup A2, distinct subhaplogroups have emerged, namely A2ab [C16291T], A2ad1 [A16175G], A2af1b1b [C16168T], A2ah [A16098G], and A2i [T16325C]. The A2ab subhaplogroup has been identified in contemporary Native American populations residing in the United States [47]. Similarly, the A2ad1 and A2af1b1b subhaplogroups have been detected in present-day population groups with indigenous ancestry in Panamá, and Ciudad de Colón and Costa Rica, respectively [46]. Conversely, the A2ah haplogroup has been observed among indigenous populations in Bolivia, with an estimated origin of approximately 5200 years ago [48]. Furthermore, this haplogroup has also been documented in Brazil [48] and northern Argentina [49,50,51].

Regarding haplogroup B2, its phylogenetic structure delineates six documented subhaplogroups: B2a, defined by [16111T] transitions [45]; B2s, characterized by [16325C] [52]; B2g1, distinguished by [16298C]; B4j, identified by [16223T]; Bac1a1a, defined by [16086C]; and B4a1c3, recognized by [16194C].

The B2a haplotype has been observed among various population groups in Mexico, southwest Honolulu, and the United States, as well as the Nuxálk, Apache, Navajo, and Tsimshian peoples [53]. Achilli et al. [45] propose that this particular haplotype emerged around 12,000 years ago in Asia. Further reassessment indicates its absence in Eskimo and Aleut populations, suggesting its emergence as a Paleoindian foundational haplotype in North America. Interestingly, the B2 haplogroup lacks ancestral clades in Siberia, although the B4b1 haplotype has been reported in Southeast Asia [54]. The phylogeography and diversity of the B2 haplogroup mirror those of A2 and align with a substantial population expansion from North America to South America.

Limited information exists regarding the population of the B2g1 subhaplogroup. According to the EMPOP database, this variant has been identified in 20 individuals typified in Mexico and San Diego. In contrast, the B4j subhaplogroup has not been documented in other populations. In Asia, the monophyletic nature of the B4 haplogroup has been consistently debated. The ancestral branch, B4a1c haplogroup, originated in East Asia 15,000 years ago [55], with the majority of these individuals migrating to Japan and South Korea. However, reports detailing this variant solely stem from Argentina, Chile, and Kyrgyzstan [56]. Similarly, studies on the B4a1a variant indicate that population groups from Asia migrated to Polynesia [54], necessitating additional reports to formulate migration models.

Confirming the presence of the B4c1a1a haplogroup presents a more intricate challenge. With no reports in the Americas and sparse occurrences in Asia and Japan, Tanaka et al. [36] were unable to locate the [16194] polymorphism. However, the EMPOP database does indicate instances of B4c1a1a in four Japanese individuals, one South Korean, and one from Hong Kong.

The C1 haplogroup is widespread among current American populations [57], suggesting the possibility that the A1, B1, C1, and D1 haplogroups colonized America in a unified migration wave [58]. C1 is considered a foundational haplogroup [41] and is notably prevalent in ancient Mayan populations [59,60]. Similarly, high frequencies of the A and B haplogroups have been observed in these populations. This pattern is also evident in the remains found at the Maya site of Copán, where the frequency of the C1 haplogroup closely resembles that found in ancient Caribbean groups [61,62]. Furthermore, there is a moderate frequency noted in ancient South American populations while, in contemporary populations, its prevalence remains high.

Lastly, the C1b5b haplogroup has been documented in seven individuals from the central region of South America, while C1C4 appears in 29 subjects from El Salvador. The D4h3a clade predominantly inhabits South America and, according to Perego et al. [63], it represents a rare variant that entered America through two potential avenues: a migration route via the Pacific Ocean and another following the coastal paths through Beringia. Regardless of the path it took to reach this continent, D4h3a is unmistakably a foundational haplogroup [64].

Drawing from the phylogeny proposed by Derenko et al. [65], D4h3a originates from European roots. However, Parson and Dür [34] report its presence in one individual from North America, one in Iran, and one in Hong Kong, suggesting a possible origin in northern Asia [66].

## 4. Discussion

The analysis of the three studied population groups reveals a strikingly elevated level of genetic diversity compared to the contemporary populations residing in Colombia [67,68]. What is even more remarkable is that this heightened genetic diversity becomes even more pronounced when juxtaposed with the findings of previous studies on ancient populations across Latin America. This compelling evidence strongly suggests that Colombia played a pivotal role as a central hub, not only for cultural interactions but also for significant biological exchanges [2,9,68,69].

This wealth of genetic diversity is not just a matter of academic curiosity; it opens up intriguing avenues of inquiry into the complex history of human populations in this region. It prompts questions about migration patterns, interactions between different groups, and the dynamic interplay between human communities and their environment.

It is important to delve deeper into the implications of this diversity. One plausible hypothesis is that Colombia, with its diverse landscapes, may have acted as a meeting point for various population groups over time. This could be attributed to its geographical location, which bridges different regions and environments within the Americas.

The concept of Colombia as a hub for cultural interactions underscores the significance of this region as a melting pot of diverse traditions, languages, and lifestyles. It becomes evident that the rich tapestry of human experiences and interactions in Colombia has deep historical roots [70].

From a biological perspective, the elevated genetic diversity observed suggests a long history of human presence in the area, marked by migrations, adaptations, and the mixing of distinct genetic lineages. This scenario paints a picture of a region where different groups have continuously crossed paths, leading to genetic intermingling and the emergence of unique genetic profiles [71].

When we examine the mitochondrial DNA (mtDNA) haplotypes found in ancient remains from present-day Colombia, we are presented with invaluable evidence that can serve as a cornerstone for constructing hypotheses about the populations that once inhabited Colombian territories. One conceivable scenario is that Amerindians, the indigenous peoples of the Americas, trace their origins back to a relatively small group that embarked on a momentous migration from Northeast Asia into the Americas, as well as adjacent regions in Western Asia. This initial migration would have marked a pivotal moment in human history, as it laid the foundation for the peopling of the entire American continent. [72,73,74]

However, the story does not end with this initial migration. It is likely that, over time, the descendants of these early migrants underwent a process of fragmentation into smaller, distinct entities. These smaller groups likely dispersed across the vast expanse of the American continent. This phenomenon of fragmentation and dispersion among these groups is a recognized genetic process known as “genetic drift”.

Genetic drift can be likened to the branching of a tree, where each branch represents a separate population group. As these groups became geographically isolated from one another, they could have developed unique genetic characteristics. Over generations, these characteristics manifested as the variety of mtDNA haplotypes that we observe within each population.

Following the arrival of European colonizers in the Americas, a tumultuous period unfolded, marked by a series of impactful events including wars, the enslavement of indigenous peoples, devastating epidemics, the mixing of different ethnic groups, and various other factors. This complex historical context likely had profound consequences on the indigenous communities in the Americas [75,76].

One of the key outcomes of this tumultuous era was the isolation and population decline experienced by many of these indigenous communities. The upheaval caused by European colonization disrupted established ways of life, led to the loss of traditional territories, and resulted in significant population losses due to violence, disease, and forced labor.

Geographical distances and natural barriers, such as mountain ranges, dense forests, and vast rivers, further compounded the isolation of different American populations. These geographical features not only hindered physical movement but also limited gene flow between isolated groups. As a result, genetic drift, the process through which genetic characteristics change in isolated populations over time, became increasingly pronounced.

The cumulative effects of these historical factors can help elucidate the remarkable inter-population diversity observed in the mtDNA haplotypes examined in this study. When we contrast this diversity with the relative genetic consistency found in the maternal line of modern-day Colombian inhabitants, it becomes evident that the genetic legacy of these historic events is still discernible today.

The swift accumulation of molecular data gathered from various American populations has substantially expanded their utility in testing alternative theories related to the colonization of the Americas. This wealth of genetic information has become a valuable resource for researchers seeking to unravel the complex history of the first settlers in the Americas.

Furthermore, the integration of genetic data into insights from other disciplines, such as archaeology and paleo-environmental studies, has yielded a more comprehensive and multi-dimensional understanding of the initial colonization of the Americas. These interdisciplinary assessment studies have played a pivotal role in corroborating and refining existing theories, while shedding light on the emergence of distinct Paleoamerican groups.

Through aligning genetic data with archaeological findings, researchers have been able to construct more accurate narratives of the past. For example, the genetic evidence can complement archaeological discoveries by providing insights into the movements, interactions, and genetic relationships among ancient populations.

Similarly, the incorporation of paleo-environmental data, such as climate records and vegetation patterns, can help contextualize genetic findings. Changes in the environment over time can influence human migration patterns and adaptation strategies, and genetic data can corroborate or challenge hypotheses regarding how these factors interplayed.

Through these collaborative efforts across scientific disciplines, our comprehension of the peopling of the Americas has become richer and more nuanced. It has not only reaffirmed the significance of ancient American populations, but has also revealed the intricate tapestry of human history, showcasing the diverse groups and cultures that thrived across the continent millennia ago.

In light of recent research, there is growing support for the hypothesis that these ancient populations might have followed a migration route along the Pacific coast as they entered the Americas [76,77,78]. This alternative route, which contrasts with the more traditional view of an inland migration through an ice-free corridor, is gaining traction due to compelling evidence from various fields of study.

The idea of a coastal migration is particularly intriguing because it has the potential to shed new light on the genetic profiles of American populations, specifically in terms of human leukocyte antigen (HLA) diversity. Coastal environments offer unique ecological niches and resources that may have influenced the genetic makeup of the populations inhabiting these regions. Consequently, the genetic diversity observed in contemporary American populations, including the diversity in HLA genes related to immune responses, could be partially attributed to the selective pressures and environmental adaptations associated with coastal living [79].

Moreover, it is important to entertain the concept of reverse migration from America to Asia at various points in history. While the prevailing narrative often focuses on migrations from Asia to the Americas, genetic evidence should also prompt us to consider the possibility of human populations moving in the opposite direction. Such reverse migrations, if supported by further research, could have implications for our understanding of the complex interactions and exchanges that occurred between the two continents over millennia [80,81,82,83,84,85].

Indeed, while the findings from studies in population genetics provide substantial support for the hypotheses discussed, reconstructing the history of these populations requires a multidisciplinary approach that spans various fields of study with a shared goal. To gain a more precise and comprehensive understanding of the complex history of these ancient populations, it is imperative to integrate data into insights from genetics, archaeology, anthropology, and other relevant disciplines.

One crucial aspect of this interdisciplinary endeavor is the detailed examination of individual haplotypes. These genetic markers can yield nuanced information about the genetic diversity, migration patterns, and relationships within and between ancient populations. By scrutinizing individual haplotypes, researchers can discern specific genetic signatures, trace migration routes, and decipher the genetic impact of various historical events.

Furthermore, establishing correlations between these haplotypes is equally vital. These correlations enable researchers to compare different hypotheses or models that have been proposed to explain the origins, migrations, and interactions of these ancient populations. By identifying consistent patterns or deviations across populations, researchers can refine their theories and construct more accurate historical narratives.

However, it is essential to acknowledge that, before meaningful comparisons can be made, a critical reevaluation of previously reported South American variants is necessary. This process involves revisiting and reanalyzing existing genetic data, ensuring their accuracy, and considering how they align with the new findings. Such a comprehensive review of the existing genetic record is fundamental to building a more robust foundation for the interdisciplinary study of these ancient populations and their remarkable history.

A potentially more accurate approach has been presented by Stoneking and Krause [86], who conducted a comprehensive genome review. They based their model on initial correlation tests performed on conflicting theories developed to reconstruct the settlement history. However, it is worth noting that these approaches face a challenge: each case has its unique characteristics and can be subject to subjective statistical analysis.

## 5. Conclusions

Following the extraction and analysis of DNA from the 33 samples sourced from ancient remains dating back to 800–1600 AP and found in the departments of Boyacá, Antioquia, and Valle del Cauca, a remarkable genetic diversity and the identification of four distinct American population haplogroups were achieved. These discoveries are poised to significantly contribute to the discourse surrounding the origins of the earliest American peoples.

The prevailing heterogeneity within the A, B, C, and D haplotypes, which has been observed across contemporary populations throughout the continent, underscores the presence of the C1 and D4h3a variants. These variants lend credence to the hypothesis proposing a rapid colonization of the Pacific coast.

Haplotypes affiliated with haplogroup B4 suggest an ancestral maternal lineage linked to West Asia among ancient American populations. However, for a comprehensive understanding of haplotypes and haplogroups, it is essential to augment genetic relationship databases with more data and reassess polymorphisms identified in prior studies. This will facilitate the identification of more direct links between human groups from diverse regions based on their ancient DNA. This endeavor holds potential significance, particularly for groups that may belong to distinct cultures originating from various corners of the world.

## Data Availability

The GenBank accession numbers for the data related to this study are: BankIt2736055: OR478541–OR478622.
